# Prognostic Significance of Lymphatic, Venous and Perineural Invasion After Neoadjuvant Chemotherapy in Patients with Gastric Adenocarcinoma

**DOI:** 10.1245/s10434-020-08389-7

**Published:** 2020-03-26

**Authors:** Benjamin L. Woodham, Jakub Chmelo, Claire L. Donohoe, Anantha Madhavan, Alexander W. Phillips

**Affiliations:** 1 Northern Oesophagogastric Unit, Royal Victoria Infirmary, Newcastle upon Tyne NHS Foundation Trust, Newcastle upon Tyne, UK; 2 School of Medical Education, Newcastle University, Newcastle upon Tyne, UK

## Abstract

**Background:**

The significance of perineural (PNI), lymphatic (LI) and venous invasion (VI) in gastric cancer patients who have received neoadjuvant chemotherapy is unclear. The aim of this study is to determine the incidence and prognostic significance of LI, VI and PNI in these patients.

**Patients and Methods:**

Consecutive patients treated with neoadjuvant chemotherapy followed by gastrectomy with D2 lymphadenectomy were reviewed. Presence of LI, VI and PNI was recorded and correlated with clinical outcomes.

**Results:**

A total of 243 patients underwent gastrectomy after neoadjuvant therapy for gastric adenocarcinoma. LI was identified in 129 (53%), VI in 107 (44%) and PNI in 116 (48%) of patients. Presence of LI (HR, 2.95, CI 1.91–4.56), VI (HR, 2.66, CI 1.78–3.98) and PNI (HR, 3.85, CI 2.49–5.95) was associated with poorer survival (all *p* < 0.001). Multivariable analysis revealed that ypT stage (HR, 1.35, CI 1.05–1.74), ypN stage (HR, 1.53, CI 1.28–1.83) and PNI (HR, 2.11, CI 1.31–3.42) were independent predictors of survival.

**Conclusions:**

LI, VI and PNI are associated with poorer survival, with PNI having prognostic significance independent of lymph node status. These factors may be useful for further prognostication, in particular when multiple factors are present, and appear especially useful for prognostic stratification in patients with no nodal involvement.

Gastric cancer is an aggressive malignancy with over 950,000 new cases reported globally, making it the third commonest cause of cancer death.^1^ In patients with potentially curative disease, treatment usually includes surgery with chemotherapy. In the UK, this is commonly administered both pre- and post-operatively as part of multimodal treatment, following the results of the MAGIC trial.^2^ Pre-operative clinical staging influences whether multimodal treatment is used and may guide prognosis. Further accuracy of staging is possible after pathological examination of the resected specimen^3^ with depth of tumour invasion and extent of lymph node involvement considered core prognostic factors that are incorporated into the TNM cancer staging systems.^4^ Other prognostic factors that have been identified in multiple cancers include lymphatic vessel invasion (LI), blood vessel or venous invasion (VI) and perineural invasion (PNI),^5^ although in gastric cancer, most large studies have only reported results from patients who had surgery alone.^6^^–^8

The use of neoadjuvant therapy adds an important additional variable to the interpretation of the pathological examination which may differ from the prognostic data based on studies of patients who did not have pre-operative chemotherapy. Whilst data have been published to aid interpretation of post-neoadjuvant TNM staging, limited data are available for interpretation of other important histological findings such as lymphatic, vascular and perineural invasion (LVPNI), factors which are not included in the current international staging system (TNM 8th edition).^9^

The aim of this study is to assess the incidence and prognostic significance of LI, VI and PNI in a consecutive series of patients with gastric adenocarcinoma who received neoadjuvant therapy followed by gastrectomy with D2 lymphadenectomy at a single institution.

## Patients and Methods

### Patient Population

A contemporaneously maintained database of all patients with adenocarcinoma of the stomach was reviewed. All patients were discussed by the multidisciplinary team. Patients were included in this study if they had received neoadjuvant chemotherapy followed by either subtotal gastrectomy with D2 lymphadenectomy or total gastrectomy with D2 lymphadenectomy at the Northern Oesophagogastric Cancer Unit, Newcastle upon Tyne between 2003 and 2016. Patients were excluded if they had received surgery with palliative intent or if they died during their admission or within 90 days of surgery. Data including baseline demographics (age, gender, stage of disease and use of neoadjuvant treatment) were prospectively recorded on a standardised proforma.

### Disease Staging

Initial staging comprised endoscopy with biopsy and computed tomography (CT) scans of thorax, abdomen and pelvis. Staging laparoscopy was performed routinely in patients thought to have locally advanced disease. Endoscopic ultrasound or positron emission tomography (PET) CT were not part of the initial staging but were performed in some cases. Patients deemed to have histologically proven locally advanced disease (cT1/2, N + or T3 + , N any) without metastasis were considered for neoadjuvant treatment followed by resection. Patients with metastasis, tumours deemed unresectable during surgery or macroscopically incomplete (palliative) resections (R2) were excluded. The current UICC TNM 8th edition was used to stage all patients.^9^

### Neoadjuvant Treatment

Multiple neoadjuvant chemotherapy regimens were used throughout the present study. The majority of patients received epirubicin and cisplatin with either 5-fluorouracil or capecitabine (ECF/ECX) (193 patients) or alternatively epirubicin, oxaliplatin and capecitabine (EOX) (9 patients) as per the MAGIC protocol.^2^ Nine patients received cisplatin and 5-fluorouracil (CF), 7 patients other combinations including 5-fluorouracil and, in 25 patients, neoadjuvant regimen was not recorded. Adjuvant treatment was planned in all patients receiving multi-modal treatment as per the MAGIC protocol.

### Surgical Technique

Resections were carried out using a standardised open approach with a radical en bloc D2 lymphadenectomy.^10^ Proximal tumours and patients diagnosed with diffuse-type disease were treated with total gastrectomy. Patients with a distal tumour where adequate clearance could be achieved received subtotal gastrectomy.

### Histopathological Analysis

Specimens were resected en bloc, and immediately post-surgery, a back-table dissection was carried out by the operating surgeon for each lymph node station. These were sent in separate containers to the pathology department. Surgical specimens were fixed for 24 h in 10% formalin before sectioning. At least four blocks with tumour and adjacent benign peri-tumoral tissue were selected for histopathological evaluation and immunohistochemical staining. When no evidence of residual macroscopic tumour was identified, the specimens were more widely sampled.

Reporting was carried out by a team of specialist gastrointestinal pathologists and followed a standardised format in line with the guidelines produced by the Royal College of Pathologists,^11^ which include tumour type and differentiation, depth of tumour infiltration and degree of tumour regression as laid out by the Mandard criteria.^12^ Number of nodes recovered and number found to have nodal metastases were documented. Presence of extracapsular invasion, LI, VI and PNI were recorded routinely. No specialised staining procedures were used to identify lymphovascular infiltration. Stage groupings followed the 8th edition of TNM staging system.^9^

### Follow-Up and Definition of Recurrence

Patients were routinely followed up for 10 years. Initial outpatient review occurred at 3-month intervals in the first year and 6-month intervals for the next 2 years; thereafter, annual review was performed unless the appointment needed expediting for clinical reasons. Disease recurrence was investigated when prompted on clinical grounds and confirmed by CT scans and/or endoscopically. Death due to any cause or last clinic review or general practitioner visit was used as the end point.

### Statistical Analysis

Statistical calculations were performed by SPSS software, version 24.0 (SPSS, Chicago, IL). Categorical data were compared using the Chi-squared or Fisher’s exact tests, and a Mann–Whitney *U* test was used to compare continuous variables. Multivariable Cox regression analysis was carried out to identify independent prognostic factors. All factors from the univariable analysis with *p* value < 0.10 were entered into the multivariable analysis. *p* values < 0.05 (two-sided) were considered statistically significant.

## Results

Between June 2003 and June 2016, 252 patients underwent either total or subtotal gastrectomy after neoadjuvant therapy for adenocarcinoma of the stomach. Multiple treatment regimens were used in the present study determined by the standard of care and recruiting trials in progress at the time of each patient’s treatment. All patients received neoadjuvant therapy, although not all completed all planned cycles. Seven patients underwent surgery with palliative intent and/or underwent palliative resections due to intraoperative findings of incurable disease and were excluded from analysis. Two patients died within 90 days (both during their index hospital admission for surgery) and were excluded. LI, VI and PNI were reported in all specimen samples.

Clinicopathological characteristics of the study population are presented in Table 1. Of the 243 patients included, 171 (70%) were male; median age was 67 years (24–81 years). Total gastrectomy was carried out in 146 patients (60%) due to proximity of tumour to gastro-oesophageal junction or because of a diffuse-type cancer. The remaining 97 patients underwent subtotal gastrectomy. All patients in the present study were diagnosed with adenocarcinoma. Clinical staging indicated that 224 patients (92%) were assessed as being cT3 or greater, with 204 (84%) suspected to have lymph node involvement (cN1 +).

**Table 1:** Clinico-pathological characteristics of patients undergoing either total or subtotal gastrectomy following neoadjuvant treatment

Number of patients	243	
Age (years)	67 (24–81)	
Gender		
Male	171	
Female	72	
Tumour location		
Distal (STG)	97	
Proximal (TG)	146	
cT stage/ypT stage		
cTx/ypT0	5 (2%)	17 (7%)
cT1/ypT1	4 (2%)	31 (13%)
cT2/ypT2	10 (4%)	78 (32%)
cT3/ypT3	135 (56%)	87 (36%)
cT4/ypT4	89 (37%)	30 (12%)
cNsStage/ypN stage		
cN0/ypN0	39 (16%)	107 (44%)
cN1/ypN1	140 (58%)	52 (21%)
cN2/ypN2	52 (21%)	37 (15%)
cN3/ypN3	12 (5%)	47 (19%)
Radicality		
R0	235	
R1	8	
Median number of resected nodes	32 (10–142)	
Median number of positive nodes	1 (0–30)	

After neoadjuvant therapy and surgical resection with D2 lymphadenectomy, pathological examination revealed that eight patients (3%) had received an R1 (microscopic presence of tumour at the resection margin) resection. A median number of 32 (10–142) nodes were resected. Overall, 117 tumours (48%) were regarded as ypT3 or worse and 136 patients (56%) had nodal involvement (ypN1 +).

### Lymphovascular Invasion (LI)

Clinicopathological characteristics of patients according to presence or absence of lymphovascular invasion are presented in Table 2. Of the 243 patients, 129 (53%) were found to have LI, which was associated with more advanced ypT and ypN categories, an increased number of positive nodes post-neoadjuvant therapy and presence of VI and PNI (*p* < 0.0001). In addition, there was an association with clinical tumour (cT) stage (*p* < 0.047).

**Table 2:** Characteristics of patients in relation to presence of lymphovascular (LI), venous (VI) and perineural invasion (PNI)

	LI not present	LI present		VI not present	VI present		PNI not present	PNI present	
			*p* value			*p* value			*p* value
Number of patients	114	129	–	136	107	–	127	116	–
Age (years)	68 (43–81)	66 (24–80)	0.31	68 (24–80)	67 (28–81)	0.26	68 (24–80)	65 (28–81)	0.005
Gender									
Male	74	97	0.080	93	78	0.55	89	82	0.917
Female	40	32		44	30		38	34	
Tumour location									
Distal (STG)	46	51	0.897	62	35	0.042	59	38	0.029
Proximal (TG)	68	78		74	72		68	78	
Pre-treatment cT stage									
cT1	2	2	0.519	4	0	**0.047**	3	1	0.316
cT2	6	4		7	3		7	3	
cT3	57	78		67	68		63	72	
cT4	45	44		53	36		51	38	
cTx/0	4	1		5	0		3	2	
Pre-treatment cN stage									
cN0	23	16	0.066	24	15	0.117	23	16	0.256
cN1	67	73		82	58		69	71	
cN2	22	30		27	25		31	21	
cN3	2	10		3	9		4	8	
Radicality									
R0	111	124	0.587	132	103	0.734	127	109	**0.008**
R1	3	5		4	4		0	8	
Number of resected nodes	32 (14–103)	28 (10–129)	0.09	32 (14–142)	30 (10–71)	0.42	31 (10–103)	32 (15–142)	0.70
Number of positive nodes	0 (0–18)	3 (0–30)	**< 0.0001**	0 (0–30)	3 (0–23)	**< 0.0001**	0 (0–6)	3 (0–30)	**< 0.0001**
Post-treatment pT stage									
ypT0	17	0	**< 0.0001**	17	0	**< 0.0001**	17	0	**< 0.0001**
ypT1	26	5		30	1		29	2	
ypT2	33	45		46	32		46	32	
ypT3	29	58		32	55		29	58	
ypT4	9	21		11	19		6	24	
Post-treatment pN stage									
ypN0	76	31	**< 0.0001**	83	24	**< 0.0001**	80	27	**< 0.0001**
ypN1	21	31		27	25		24	28	
ypN2	13	24		18	19		15	22	
ypN3	4	43		8	39		8	39	
Lymphovascular invasion present	–	–	–	43	86	**< 0.0001**	40	89	**< 0.0001**
Venous invasion present	21	87	**< 0.0001**	–	–	–	28	79	**< 0.0001**
Perineural invasion present	27	89	**< 0.0001**	37	79	**< 0.0001**	–	–	–

Values are median (range). Significant *p* values in bold

### Venous Invasion (VI)

Clinicopathological characteristics of patients according to presence or absence of venous invasion are presented in Table 2. Of the 243 patients, 107 (44%) were found to have VI, which was associated with more advanced ypT and ypN categories, an increased number of positive nodes post-neoadjuvant therapy and presence of LI and PNI (all *p* < 0.0001). There was also an association of patients undergoing total gastrectomy being more likely to have VI rather than those patients undergoing subtotal gastrectomy (*p* < 0.042).

### Perineural Invasion (PNI)

Clinicopathological characteristics of patients according to presence or absence of perineural invasion are presented in Table 2. Of the 243 patients, 116 (48%) were found to have PNI, which was associated with more advanced ypT and ypN categories, an increased number of positive nodes post-neoadjuvant therapy and presence of LI and VI (*p* < 0.0001). There was also an association of patients undergoing total gastrectomy being more likely to have PNI rather than those patients undergoing subtotal gastrectomy (*p* < 0.029).

### Survival Analysis

**Fig. 1:**
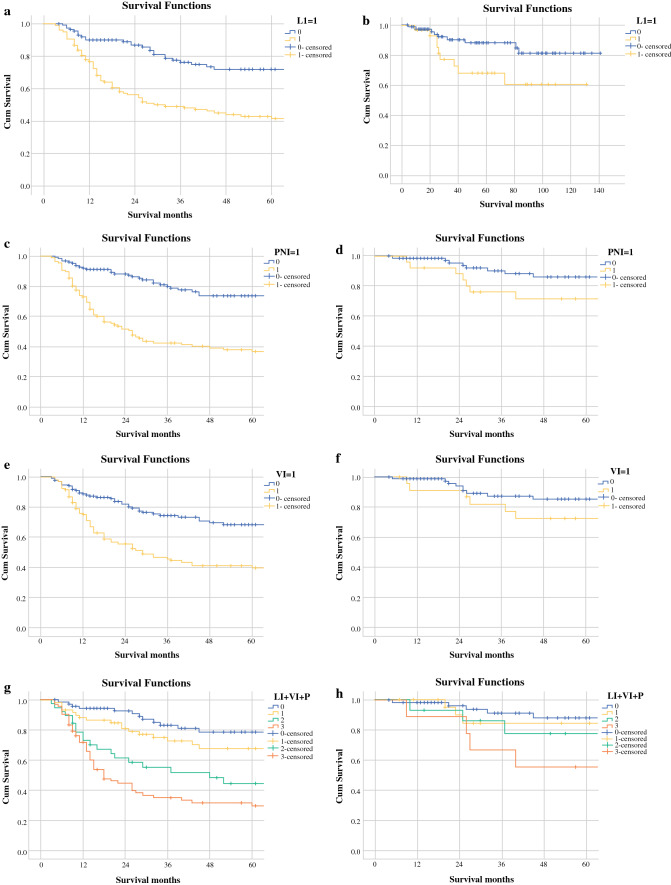
Survival curves of each factor and corresponding N0 disease: **a** lymphatic invasion (*p* < 0.001), **b** lymphatic invasion in N0 disease (*p* = 0.033), **c** perineural invasion (*p* < 0.001), **d** perineural invasion in N0 disease (*p* = 0.04), **e** venous invasion (*p* < 0.001), **f** venous invasion in N0 disease (*p* = 0.169), **g** number of factors present (*p* < 0.001) and **h** number of factors present in N0 disease (*p* = 0.038)

Death irrespective of cause was regarded as the primary outcome measure. Kaplan–Meier plots for overall survival according to ypN stage are shown in Fig. 1. Survival curves comparing when each of LI, VI and PNI were or were not present are shown in Fig. 1, alongside comparable graphs of the N0 disease cohort. Survival curves illustrating the effect of cumulative presence of one, two and three invasion factors are shown in Fig. 1g, h. Results of univariable and multivariable analyses (with respect to survival) are presented in Table 3.

**Table 3:** Univariable and multivariable analyses of factors influencing survival

	Univariable			Multivariable		
	HR	CI	*p* value	HR	CI	*p* value
Male	0.852	0.548–1.323	0.852	N/A		
Age (< 65 years vs. > 65 years)	1.01	0.99–1.03	0.318	N/A		
Operation (total vs. subtotal gastrectomy)	1.575	1.04–2.385	**0.032**			0.137
ypT	1.905	1.544–2.351	**< 0.001**	1.353	1.054–1.738	**0.018**
ypN	1.861	1.581–2.19	**< 0.001**	1.526	1.277–1.825	**< 0.001**
Radicality (R0 vs. R1)	3.273	1.426–7.51	**0.005**			0.14
Lymphovascular invasion	2.947	1.905–4.558	**< 0.001**			0.324
Venous invasion	2.66	1.779–3.978	**< 0.001**			0.782
Perineural invasion	3.846	2.486–5.95	**< 0.001**	2.113	1.306–3.419	**0.002**
Tumour regression grade	1.291	0.967–1.724	0.083	N/A		

Bold values are statistically significant

Univariable analysis demonstrated that patients in our study had poorer overall survival following subtotal rather than total gastrectomy (HR, 1.575, CI 1.04–2.385, *p* = 0.032). Survival decreased as ypT stage increased (HR, 1.905, CI 1.544–2.351, *p* < 0.001) or ypN stage increased (HR, 1.861, CI 1.581–2.19, *p* < 0.001). Survival was poorer in the small number of patients who received R1 resection (HR, 3.273, CI 1.426–7.51, *p* = 0.005). Presence of LI, VI and PNI was associated with worse survival outcomes (LI: HR, 2.947, CI 1.905–4.558; VI: HR, 2.66, CI 1.779–3.978; PNI: HR, 3.846, CI 2.486–5.95; all *p* < 0.001), and the effect was cumulative, as seen on the survival curves in Fig. 1. Multivariable analysis revealed that ypT stage, ypN stage and PNI were all independent predictors of survival (ypT: HR, 1.353, CI 1.054–1.738, *p* = 0.018; ypN: HR, 1.526, CI 1.277–1.825, *p* < 0.001; PNI: HR, 2.113, CI 1.306–3.419, *p* = 0.002).

The impact of presence of LI, VI and PNI was evaluated for those patients with no histopathological evidence of lymph node involvement (ypN0, Fig. 1). This demonstrated 5-year survival of 88% for those with zero invasion factors present (LI, VI or PNI). This dropped to 84% with one factor present, 72% with two factors present and 56% when all three were present.

### Adjuvant Treatment

Fifty-two patients received at least one course of post-operative therapy. There was no significant difference in survival between the two cohorts. Five-year survival was 57% for those not receiving adjuvant treatment compared with 56% for those who received at least one cycle (*p* = 0.747).

## Discussion

Gastric cancer is an aggressive malignancy in which most patients treated with surgical resection develop disease recurrence, with a recent meta-analysis showing that 5-year survival for patients undergoing surgery is 42% if given neoadjuvant therapy and only 30% if not.^13^ The present study indicates that presence of LI, VI and/or PNI is associated with poorer survival in patients with gastric adenocarcinoma who are treated with neoadjuvant therapy followed by gastrectomy with D2 lymphadenectomy, with PNI having prognostic significance independent of lymph node status. The reported prevalence of LI, VI and PNI is widely distributed, with PNI being reported between 2% and 48%, VI 7–44% and LI 10–91%. However, these values correspond to chemotherapy-naïve patients.^14^^–^16

Patients with gastric cancer who undergo surgery are staged by the TNM system. The importance of the number of involved nodes is reported in the 7th^4^ and 8th^9^ editions of the TNM system, and our data reflect this, as seen in Fig. 1. Pre-treatment clinical staging guides prognostication and choice of treatment options that are discussed with patients; however, our analysis demonstrates minimal association between clinical staging and presence of LI, VI and PNI. There is an association between ypT and ypN stage and LI, VI and PNI post-neoadjuvant therapy. A combination of these factors is associated with poorer survival, an important finding given that this study demonstrates that presence of one form of invasion increases the risk of other forms being present. This study suggests that presence of LI, VI and/or PNI in the surgical specimen is an indicator for aggressive disease behaviour that should be taken into consideration along with ypT and ypN stage. Evaluation of survival curves in those with N0 disease demonstrated similar, albeit less pronounced, outcomes to those in the entire cohort. Whilst a statistical significance remains with each factor (except VI), the change in significance may be due to the smaller dataset of these patients. The present study provides an insight into the survival outcomes associated with LI, VI and PNI in a large series of gastric adenocarcinoma patients who have undergone neoadjuvant therapy followed by surgery. Many previous studies have reported that LI, VI and PNI are adverse prognostic factors in other cancers, such as in colorectal,^17^ gynaecological,^18^ breast^19^ and pancreatic^20^ cancers. In gastric cancer, LI,^21^ VI^22^ and PNI^23^ have all been reported as adverse prognostic factors, although this is predominately in patients who have not received neoadjuvant therapy. Jhawer et al.^24^ reported that perineural invasion was associated with worse outcomes in a study of 38 post-neoadjuvant patients, and Zhu et al.^25^ reported LI and PNI (but not VI) data for 192 post-neoadjuvant patients, showing worse survival if one factor was present, but with no assessment of the additive risk of multiple factors. The present study confirms and expands on these previous findings by establishing that LI, VI and PNI are individually associated with poorer survival outcomes, with PNI acting independently of other predictors of survival. The present study also demonstrates that survival worsens as the total number of these histopathological factors increases. This is in good agreement with what has been seen in oesophageal adenocarcinoma.^5^

In addition, the association between combined LVPNI and survival occurs even in patients with no tumours detected in the surgically resected lymph nodes (ypN0 subgroup). Patients in this subgroup have substantially better survival than those with even small numbers of positive nodes. In the present study, 44% of patients had no residual nodal disease, and our analysis of this subgroup suggests that survival outcomes can be further stratified using LVPNI (Fig. 1). The present study demonstrates that, in the ypN0 subgroup, presence of multiple LVPNI factors (despite no positive nodes) is associated with significantly worse survival outcomes. Patient compliance with adjuvant therapy is often poor, and multi-disciplinary teams may find the additional prognostic information provided by LVPNI factors to be useful in encouraging take-up of adjuvant therapy, especially in what might otherwise be considered low-risk disease on a purely TNM basis.

Presence of LVPNI may be useful when stratifying patient populations in future studies assessing the efficacy of adjuvant regimens. These factors also have prognostic potential in pre-operative patients for whom this information exists, for example in patients who have had endoscopic mucosal resection biopsies^22^,^26^,^27^ or in those whose simple biopsies happen to include evidence of LVPNI. Further, these factors may help identify patients with poorer prognosis. In those for whom neoadjuvant treatment has had apparently little impact (e.g. TRG 4/5), it may help support a different adjuvant modality such as chemoradiotherapy. However, this highlights a potential area for future work.

In conclusion, these findings suggest that presence of LI, VI and PNI after neoadjuvant therapy followed by gastrectomy is associated with poorer prognosis. These factors should be incorporated in standard pathology reports and should be considered by multi-disciplinary teams when identifying patients at higher risk of disease recurrence or when considering the need for adjuvant therapy. This may be particularly useful in decision-making, when multiple factors are present, for node-negative patients who otherwise might be considered relatively low risk for recurrence.
